# Prevalence of Depression in a Large Urban South Indian Population — The Chennai Urban Rural Epidemiology Study (Cures – 70)

**DOI:** 10.1371/journal.pone.0007185

**Published:** 2009-09-28

**Authors:** Subramani Poongothai, Rajendra Pradeepa, Anbhazhagan Ganesan, Viswanathan Mohan

**Affiliations:** Madras Diabetes Research Foundation & Dr. Mohan's Diabetes Specialities Centre, WHO Collaborating Centre for Non-Communicable Diseases -Prevention and Control, Gopalapuram, Chennai, India; University of Cape Town, South Africa

## Abstract

**Background:**

In India there are very few population based data on prevalence of depression. The aim of the study was to determine the prevalence of depression in an urban south Indian population.

**Methods and Findings:**

Subjects were recruited from the Chennai Urban Rural Epidemiology Study (CURES), involving 26,001 subjects randomly recruited from 46 of the 155 corporation wards of Chennai (formerly Madras) city in South India. 25,455 subjects participated in this study (response rate 97.9%). Depression was assessed using a self-reported and previously validated instrument, the Patient Health Questionnaire (PHQ) – 12. Age adjustment was made according to the 2001 census of India. The overall prevalence of depression was 15.1% (age-adjusted, 15.9%) and was higher in females (females 16.3% vs. males 13.9%, p<0.0001). The odds ratio (OR) for depression in female subjects was 1.20 [Confidence Intervals (CI): 1.12–1.28, p<0.001] compared to male subjects. Depressed mood was the most common symptom (30.8%), followed by tiredness (30.0%) while more severe symptoms such as suicidal thoughts (12.4%) and speech and motor retardation (12.4%) were less common. There was an increasing trend in the prevalence of depression with age among both female (p<0.001) and male subjects (p<0.001). The prevalence of depression was higher in the low income group (19.3%) compared to the higher income group (5.9%, p<0.001). Prevalence of depression was also higher among divorced (26.5%) and widowed (20%) compared to currently married subjects (15.4%, p<0.001).

**Conclusions:**

This is the largest population-based study from India to report on prevalence of depression and shows that among urban south Indians, the prevalence of depression was 15.1%. Age, female gender and lower socio-economic status are some of the factors associated with depression in this population.

## Introduction

Depression is an illness that affects both the mind and the body and is a leading cause of disability, workplace absenteeism, decreased productivity and high suicide rates [1], [2]. Depression is the most common psychiatric disorder in general practice and about one in ten patients seen in the primary care settings suffer from some form of depression [3], [4]. In a study by the World Health Organization (WHO) conducted at 14 sites, the most common diagnosis in primary care was depression [5].

Depression is estimated to affect 340 million people globally [6]. The prevalence of psychiatric disorders is reported to differ between countries and within countries, across various ethnicities [7]. Most studies on depression are from the developed world and there are few studies from developing countries. The World Mental Health Survey Initiative [8] carried out cross-national research in mental health, especially in developing countries. The prevalence of depression in a population based study conducted in urban Pakistan was 45.9% [9], while in rural Bangladesh, it was reported to be 29% [10] and in a peri-urban clinic based study in Uganda, it was reported to be 6.1% [11].

Earlier Indian studies have reported prevalence rates of depression that vary from 21–83% in primary care practices [12–15.] There have been a few population based studies from India but most have been done on selected groups [16]–[21]. We are not aware of a study on depression from India covering a whole city with all adult age groups included. This paper reports on the prevalence of depression in an urban south Indian population and, is to our knowledge, the largest study on depression from India till date.

## Materials and Methods

The subjects for this study was recruited from the urban component of the Chennai Urban Rural Epidemiological Study (CURES), conducted on a representative population of Chennai (forming Madras city) in South India. The methodology of CURES has been published elsewhere [22] and the sampling frame is available at http://www.drmohansdiabetes.com/mdrf/CURES.pdf. Briefly, the whole of Chennai has been divided into 10 zones and 155 wards by the Chennai Corporation representing a socio-economically diverse group. The sampling for CURES was based on the model of systematic random sampling, wherein, of the 155 wards, 46 wards were selected to represent all the 10 zones. The total sample size of 26,001 individuals was selected from these 46 wards. The sample distribution in each ward within these zones is based on the proportion of their population in that particular zone. Further, within each ward, every third lane or road, following the right hand rule was surveyed. Such a sampling approach was chosen as it enabled the arrival of an equitable distribution of the entire Chennai population while ensuring that the sampling error is kept to a minimum. Another advantage is the simplicity of the administrative procedures involved. All men and women≥20 years of age were considered eligible for the study. Phase 1 of CURES was conducted from June 2001 to August 2002, in the field, and involved a door-to-door survey in the selected wards. Self-reported diabetic subjects on drug treatment of diabetes were classified as known diabetic subjects. The study was approved by the Institutional Ethics Committee of the Madras Diabetes Research Foundation and written informed consent was obtained from all participants before participating in the study.

In all participants, a baseline home visit was made and a questionnaire was administered to assess the demographic and socioeconomic characteristics, health behaviours and health status including a detailed medical history. Details obtained included the participant's self-reported age, sex, years of education, family income. Fasting capillary blood glucose was determined using a hand held glucose monitor (One Touch Basic, Lifescan Johnson & Johnson, Milpitas, California, USA) in all subjects. Anthropometric measurements including weight, height and waist measurements were obtained using standardized techniques as described earlier [23]. The body mass index (BMI) was calculated using the formula, weight (kg)/height (in meters squared). Blood pressure was recorded in the sitting position in the right arm to the nearest 2 mmHg with a mercury sphygmomanometer (Diamond Deluxe BP apparatus, Pune, India). Two readings were taken 5 min apart and the mean of the two was taken as the blood pressure.

A widely and internationally used instrument, the Patient Health Questionnaire (PHQ) - 9 item [24], [25] was used but modified for local conditions into a 12 item questionnaire (PHQ-12 item, Table 1). The modified -12 item was developed with yes or no response by using the same version of PHQ-9 item; however, three of the questions in the PHQ-9 item were split into two and thus accounting for 12 items. The response categories were also made dichotomous (yes/no), such that the subject would be asked whether they had any of the twelve depressive symptoms and, if yes, their frequency, during the last two weeks. The reason for making the response as yes or no in the modified PHQ-12 item was because it was easier to screen depression in large epidemiological studies. The modified PHQ-12 item was then validated in the same population and shown to be a reliable instrument for screening of depression in the general population with a cut-off score of greater than 4 being the best predictor of depression with a sensitivity of 92.0% and specificity of 90.7% using PHQ-9 as the gold standard [26]. It took 10 minutes to administer the questionnaire which was in the English language.

**Table 1:** Modified PHQ-12 questionnaire.

Modified Patient Health Questionnaire (PHQ) -12 item
***Response:*** * Yes/No*
1. Feeling sad, blue or depressed
2. Loss of interest or pleasure in most things
3. Feeling tired or low on energy most of the time
4. Loss of appetite or weight loss
5. Overeating or weight gain
6. Trouble falling asleep or staying asleep
7. Sleeping too much
8. More trouble than usual concentrating on things
9. Feeling down on yourself, no good, or worthless
10. Being fidgety or restless that you move around a lot more than usual
11. Moved or spoke so slowly that other people could have noticed
12. Thought about death more than usual, either your own, someone else's, or death in general
***Scoring System*** *:1 point for every ‘yes’*
*Depression defined as score>4 based on validation study * [***26***]

All statistical analyses were performed using SAS for window version 9.0 software (SAS Institute Inc, Cary, NC, USA). Numbers are expressed as mean±standard deviation. Student's “t” test was used to compare groups for continuous variables. Chi square test was used to compare proportions among groups. The prevalence rate obtained in the present study was age-standardized to the 2001 Census of India using direct method. Univariate logistic regression analysis was performed using variables which are known to be associated with depression such as marital status, education and income to look at their association with depression in our population. The dependent variable was dichotomized as no depression = 0 and depression = 1. Categorical independent variables were scored as follows:: Marital status [unmarried = 0 (reference), married = 1, widowed = 2 and divorced = 3] for both men and women; education status [professional = 0 (reference), post-graduate = 1, graduate = 2, secondary school certificate = 3, below secondary school certificate = 4 and Illiterate = 5]; income level in Indian Rupees [>20000 = 0 (reference), 10001–20000 = 1, 5000–10000 = 2 and <5000 = 3]. For all statistical tests, p value<0.05 was considered as the level of significance.

## Results

Of the 26,001 individuals who participated in the CURES study, 25,455 subjects participated in this study (response rate: 97.9%) which included 12,557 males (49.3%) and 12,898 females (50.6%). The mean age of the study population was 39±14 years.

The prevalence of depression in the study population is presented in Table 2. The overall crude prevalence of depression was 15.1% [95% Confidence Interval (CI):14.7–15.6] and the age-standardized prevalence, adjusted to the 2001 population of Chennai, was 15.9% (95% CI: 15.6–16.5). Female subjects had higher crude prevalence of depression compared to male subjects [females: 16.3% vs. males: 13.9%, p<0.001] and the respective age-standardized prevalence rates were 17.4% (females) and 14.5% (males, p<0.001). The odds ratio (OR) risk for depression in female subjects was 1.20 (CI- 1.12–1.28, p<0.001) compared to male subjects.

**Table 2:** Prevalence of Depression in the Chennai Study Population.

Subjects (n)	Subjects with	Crude prevalence	Age Standardized a prevalence
	Depression	% (95% CI)	% (95% CI)
**Total (n = 25455)**	3847	15.1(14.7–15.6)	15.9 (15.6–16.5)
**Male (n = 12557)**	1750	13.9 (13.3–14.6)	14.5 (13.4–14.6)
**Female (n = 12898)**	2097	16.3*(15.3–16.9)	17.4 (16.4–17.7)

a Direct age standardization based on 2001 Chennai census.

CI- Confidence Interval; * p<0.001 compared to male gender.

The clinical and biochemical characteristics of the study groups with and without depression are shown in Table 3. Subjects with depression were older (p<0.001) and had higher waist circumference (p<0.001), fasting capillary glucose (p<0.001), systolic blood pressure (p<0.001) and diastolic blood pressure (p<0.0001) compared to those without depression.

**Table 3:** General characteristics of the study subjects.

Variables	Subjects without	Subjects with	P value
	depression	depression	
	(n = 21608)	(n = 3847)	
Age (years)	37.2±13.6	43.2±15.9	<0.001
Men n (%)	10807 (50.0)	1750(45.5)	<0.001
Height (cms)	160.2±8.3	159±8.1	<0.001
Weight (kgs)	57.6±10.7	56.4±10.8	<0.001
Waist circumference (cms)	79.5±10.8	80.1±11.4	<0.001
Body mass index (kg/m^2^)	22.5±4.1	22.5±4.4	0.474
Fasting capillary glucose(mg/dl) [mmol/L]	101±32 [5.6]	106±41 [5.9]	<0.001
Systolic BP (mmHg)	118±17	120±20	<0.001
Diastolic BP (mmHg)	79.9±10.4	80.5±10.7	<0.001
Smoking n (%)	1867 (8.7)	364 (9.5)	0.094
Alcohol n (%)	1226 (5.7)	230 (6.0)	0.455

The various symptoms of depression found in the study population are presented in Table 4. Depressed mood was the most common symptom (30.8%) followed by tiredness (30.0%), while more severe symptoms such as suicidal thoughts and speech and motor retardation were less common (12.4%).

**Table 4:** Symptoms of depression reported by study subjects.

Symptoms	(%) of ‘yes’ responses
Depressed mood	30.8
Tiredness	30.0
Loss of interest	29.7
Trouble falling sleep or staying asleep	25.1
Lack of concentration	21.1
Sleeping too much	19.8
Loss of appetite or weight loss	19.1
Over eating or weight gain	17.7
Feeling of worthlessness	16.8
Being fidgety or restless	14.5
Suicidal thoughts	12.4
Moved or spoke slowly	12.4

**Note**: Numbers do not add up to 100% as some subjects reported more than 1 symptom.

**Figure 1:**
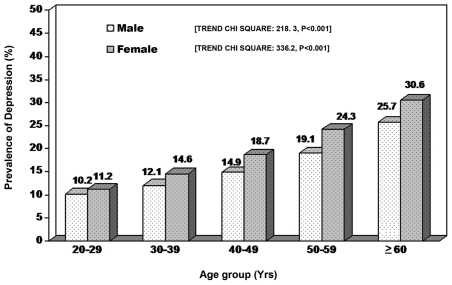
Gender and age-wise prevalence of depression in Chennai.

Age wise prevalence of depression in both genders is shown in Figure 1. With increasing age, there was an increasing trend in the prevalence of depression among both female (Trend χ2: 336.2, p<0.0001) and male subjects (Trend χ2: 218.3, p<0.0001).The prevalence of depression among female subjects was significantly higher compared to their male counterparts at all age groups

Table 5 presents the prevalence of depression with respect to socio-economic parameters. The data shows that an inverse relationship was observed between household income and depression rates, with prevalence of depression being higher in the low income group [income<Rs.5000/month, $ 100] −15.7% compared to the higher income group [income ranging between >Rs.20,000/month,$400] −7.1% (Trend χ2 70.3, p<0.001). Individuals with lower low levels of education were more depressed compared to those who were better educated (Trend χ2: 410.1, p<0.001).

**Table 5:** Univariate logistic regression analysis of depression with marital status, education and income levels.

Parameters	Prevalence of	Odds Ratio (95% CI)	P value
	Depression (%)		
**Marital status [Men]**			
Unmarried(reference)	10.4	1.000	
Married	14.9	1.51(1.32–1.70)	<0.001
Widowed	29.1	3.52(2.61–4.76)	<0.001
Divorced	7.7	0.72(0.22–2.33)	0.578
**Marital Status [Women]**			
Unmarried (reference)	10.5	1.000	
Married	15.9	1.61 (1.37–1.88)	**<0.001**
Widowed	26.6	3.10 (2.56–3.73)	**<0.001**
Divorced	20.0	2.13 (1.01–4.49)	**0.047**
**Education status**			
Professional(reference)	6.7	1.000	
Post graduate	7.3	1.09 (0.52–2.31)	0.811
Graduate	8.9	1.35 (0.70–2.59)	0.365
SSC	12.6	2.00 (1.05–3.82)	**0.035**
Below SSC	16.1	2.66 (1.40–5.07)	**0.003**
Illiterate	25.6	4.77 (2.50–9.11)	**<0.001**
**Income level ( Rs)**			
>20000 (reference)	7.1	1.000	
10001–20000	8.1	1.15 (0.54–2.43)	0.715
5000–10000	9.7	1.41 (0.70–2.81)	0.337
<5000	15.7	2.44 (1.24–4.81)	**0.010**

(1 USD = Rs. 50 approximately)

Univariate Logistic regression analysis model was performed using depression (0 = non-depressed and 1 = depressed) as the dependent variable and variables known to be associated with depression such as marital status (separately for men and women), educational status and income level as independent variables as shown in Table 5. Taking “unmarried” as reference, the odds ratios for the married, widowed and divorced males were 1.51 (p<0.001), 3.52 (p<0.001) and 0.72 (p = 0.578) respectively. In females, the corresponding odds ratios were married- 1.61 ( p<0.001), widowed - 3.10 (p<0.001) and divorced - 2.13 (p<0.047) respectively.

When educational status was taken as the independent variable with professionals as the reference group, the odds for high school, below high school and illiterate were 2.00 (p = 0.035), 2.66 (p = 0.003), and 4.77 ( p<0.001) respectively.

When income was taken as the independent variable and higher income group as reference, the odds ratio for lower income group (i.e., income<Rs.5000) was 2.44 (p<0.010).

## Discussion

This is the largest study to our knowledge, to report on the prevalence of depression from India and the first in a representative population of adults of all ages of a large metropolitan city. We report that the crude prevalence of depression is 15.1%. This is consistent with the figures reported for developing countries [10–44%] by the WHO [7]. In a community-based study conducted among adult women belonging to fisherman community in Karachi, Pakistan, the prevalence of depression was 7.5% using Mini International Neuropsychiatric Interview, supplemented by ICD-10 [27]. Depression was reported in 9.1% of individuals in Bangalore city in South India in the WHO study done in 15 primary care centers [5].

Table 6 compares the prevalence of depression in different populations [9], [19]–[21], [28]–[32]. We only included the studies that were population based and from an urban area. It is seen that the estimates on prevalence of depression vary widely in different populations, which could be attributed to different ethnicity and demography of the study populations and different diagnostic criteria and study instruments employed.

**Table 6:** Studies on prevalence of depression in urban areas in population based studies.

Author/Year	Place	Total subjects	Age (years)	Diagnostic Criteria	Population	Method of Survey	Prevalence of Depression (%)
Rajala et al, 1994^28^	Finland	1008	>55	Zung Self Rating Scale	Elderly population	Self-reported survey	9.5
Chandran et al, 2002^ 19^	Vellore [India]	359	22.2	ICD-10 and( CIS-R),a Revised Clinical Interview Schedule	Rural women of low income population	Structured interview	11
Patel et al, 2002 ^20^	Goa [ India]	270	Mean age-26	General Health Questionnaire	Rural women of low income population	Structured interview	13
Olsen et al, 2004^29^	Hilleroed [Denmark]	2040	20–79	Major Depression Inventory (MDI), a validated self-rating scale fulfillingthe symptomatic criteria in DSM-IV &ICD-10 for a depressive episode.	Danish General population	Self - reported survey	3.3
Aluoja et al, 2004^30^	Tartu [Estonia]	4677	15–79	Emotional State Questionnaire (EST-Q), a self-rating scale of depression & anxiety	Estonian population	Structured interview	11.1
Ovuga et al, 2005 ^31^	Adjumani & Bugir [Uganda]	939	≥18	13 item Beck Depression Inventory(BDI)	Rural population	Structured interview	17.4
Muhammad, Gadit et al 2006 ^9^	Lahore, Quetta and Karachi [Pakistan]	820	Mean age - 35	Depressive symptom questionnaire	House-hold population	Telephone survey	45.9
Vasiliadis et al, 2007^32^	Canada & USA	3,505 5,183	≥18	Diagnostic and Statistical Manual of Mental Disorders (DSM-IV)	Urban population	Telephone survey	8.2 8.7
Biswas et al, 2009 ^21^	Vellore, [India]	204	>60	(CIS-R),a Revised Clinical Interview Schedule	Elderly population	Door to door survey	31.5
**Present study**	**Chennai [ India]**	**25,455**	**≥20**	**Modified Patient Health Questionnaire (PHQ-12 item)**	**Representative sample of Chennai**	**Door to Door survey**	**15.1**

In a systematic review, Mirza and Jenkins [33] reported that the major factors associated with depressive disorders were female sex, middle age, low level of education, financial constraints and relationship problems. In this study we have looked into these factors and found that there was an increasing trend in the prevalence of depression with increasing age in both genders. Stordal et al [34] in a large population based study observed that there is a linear rise with age of self reported symptoms of depression. This may be attributed to age- related decline in central serotonergic function, which might make older individuals more vulnerable to depression [35]. In a study conducted in 1992 in Illinois in USA [36], it was reported that depression reached its lowest level in the middle age at about age 45, with a rise in later life [>80 years], which reflected life cycle gains and losses related to marriage, employment and economic well being. Our study did not show any decline in the middle age, instead there was a steady increasing trend observed with age. This might be due to the cultural or socioeconomic differences between USA and India. It is possible that India being a developing country and there being an association between depression and lower socio-economic status, the prevalence of depression is seen across the lifespan. It could also be explained by cultural differences. In India, the joint family system was in vogue till recently. This provided social security to younger individuals. The break down of the joint family and the emergence of the nuclear family could explain the occurrence of depression at younger ages due to reduced family support.

Several large epidemiological studies have shown that women have higher depression rates than men [37], [38]. A meta-analysis of studies conducted in various countries has shown that women are roughly twice as likely as men to experience or report depression [39]. In our study also, women had higher prevalence rate of depression, which is consistent with earlier studies [40], [41].However it is of interest that another study done in Bangalore among young adults attending college, men were found to be more depressed (25%) than women (18%) [42].

Some studies have shown that economic hardship is a significant cause of depression. Our data showed that there was an inverse relationship in prevalence of depression with income and education. It should be pointed out that this is only an association and not a causal relationship. However, this is consistent with western studies, where people in the lower economic status were reported to be more depressed compared to those in the middle and high income status [43], [44]. It has also been shown that illiterate people have higher prevalence of depression compared to their more educated counterparts [45]. The relationship of depression with divorce and being widowed was an expected result as reported earlier in USA [36] and Finland [46] as bereavement, divorce and separation are well known causes of depression. In a population based study from Pakistan [9], the prevalence of depression was reported to be unusually high and the reasons quoted by the authors were the following: local cultural influences, geographical location and social adversities.

Table 3 shows that those with depression were older, had higher blood pressure and fasting capillary glucose levels and waist measurements. This lends weight to the maxim “no health without mental health” as argued in a recent review [47]. It is clear from this study that subjects with comorbidities have a higher prevalence of depression. This emphasizes the fact that depression or indeed any mental illness should not be considered or treated in isolation. A wholistic approach to health care looking at both physical and mental health is required if we are to tackle the problem of depression in the community.

With reference to the individual symptoms, we found the percentage of subjects with suicidal thoughts is higher (12.4%) in our study compared to other studies (3.1–9.2%) [48]–[50]. This could be because the latter have looked only at suicidal plans (or) ideation whereas our question includes suicidal thoughts or thoughts about death in general. Some participants with recent bereavement could have responded “yes” to this question and this could account for the differences observed in the response rates to this question.

The strengths of this study are that it is the largest population based [n = 26,001] done in a representative population of a large metropolitan city with a population of 6 million and used a validated study tool. One of the limitations of the study is that the prevalence of depression was based on self-reported data, which may be subject to recall bias and cultural factors. Another limitation is that being a cross sectional study, no cause effect relationship can be determined as this would need a longitudinal study. The third limitation is that as a multivariate analysis was not done, there could be potential confounding factors which could affect the results. Finally while the data may be extrapolated to urban India, it may not be relevant to rural India where 70% of Indians live, as life style and socio-cultural factors vary widely in urban and rural areas.

In summary, we report that the overall prevalence of depression in Chennai city in south India was 15.1% and that female gender, age, low socio-economic status, lack of education and marital factors are associated with depression in this population. Therefore there is a clear need to increase mental health services and to integrate this with general health services in India. Moreover, the health policy needs to address the issue of depression with particular reference to the important socio-economic risk factors for depression such as income deprivation and lack of education.
